# Mechanical Properties of Aluminum Alloy Tubes Fabricated Through Surface Mechanical Grinding Treatment and Graphene Lubrication Under Biaxial Stress States

**DOI:** 10.3390/ma18092038

**Published:** 2025-04-29

**Authors:** Yang Cai, Xiao-Lei Cui, Chunhuan Guo, Fengchun Jiang, Piaoping Yang

**Affiliations:** 1 Key Laboratory of Superlight Materials & Surface Technology, Ministry of Education, College of Materials Science and Chemical Engineering, Harbin Engineering University, Harbin 150001, China; 2 School of Materials Science and Engineering, Heilongjiang University of Science and Technology, Harbin 150022, China; 3 National Key Laboratory of Precision Hot Processing of Metals, Harbin Institute of Technology, Harbin 150001, China

**Keywords:** mechanical properties, aluminum alloy tube, surface mechanical grinding treatment, biaxial stress state

## Abstract

To enhance the mechanical properties of 6063-T4 aluminum alloy tubes, surface mechanical grinding treatment was conducted under graphene-assisted lubrication. The effects of rotational speed and cooling conditions on the mechanical properties of aluminum alloy tubes under biaxial stress were systematically explored. It was found that increasing the rotational speed and cooling rate facilitates the formation of finer lamellar grains, higher-density nano-precipitates, and a reduced dislocation density on the tube surface. These microstructural characteristics significantly contribute to an increased yield strength and sustained strain hardening capacity during bulging deformation. This study proposes an innovative approach for improving the strength and toughness of light alloy components during integral forming, providing meaningful insights for future engineering applications.

## 1. Introduction

Aluminum alloy tubular components with integral structures are extensively utilized in major aerospace and automotive structures due to their lightweight nature and high reliability [1]. Rocket fuel delivery pipelines are fabricated using 5A03 aluminum alloy tubes, while automobile subframes are hydroformed from 6063 aluminum alloy tubes [2]. These applications achieve weight reductions exceeding 40% and significantly enhance reliability compared to traditional steel welded parts. However, the relatively low strength of aluminum alloys and their propensity to develop orange peel defects during plastic deformation pose significant challenges to the broader application of integrally formed aluminum alloy tubular components. Since the “orange peel” defects can serve as crack initiation sites under complex loading conditions, they can lead to potential cracking. These defects result from surface roughening, which is closely associated with surface grain size. Studies have shown that no surface orange peel defects form when the average grain size remains below 105 μm [3]. Consequently, reducing the surface grain size represents an effective strategy to mitigate “orange peel” defects.

**Figure 1:**
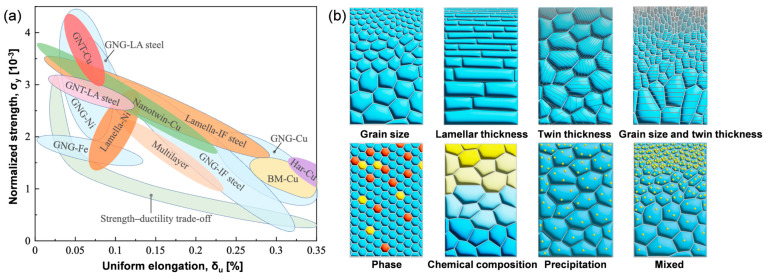
(**a**) An Ashby map of normalized strength versus uniform elongation for gradient-structured metals and (**b**) alloys and structural and chemical gradients in typical metals and alloys [10].

The Surface Mechanical Grinding Treatment (SMGT) can induce the formation of nanograins on the surface of rotating components [4], effectively preventing the occurrence of “orange peel” defects. This treatment leads to a continuous nano-gradient structure within the metal, characterized by variations in grain size, dislocation density, and second-phase distribution [5]. Consequently, the material exhibits significantly enhanced yield strength [6], achieving an improved balance between strength and ductility (as shown in Figure 1). For instance, SMGT of a copper rod resulted in a grain size gradient ranging from 20 nm at the surface to 300 nm at the core, doubling the yield strength while preserving comparable tensile ductility relative to coarse-grained copper rods [7]. Similarly, a Cu-4.5Al alloy with a gradient microstructure featuring nanotwins and elevated dislocation density demonstrated nearly double the yield strength with only a minor reduction in uniform elongation [8]. Furthermore, researchers have applied SMGT to machine tool spindles, resulting in enhanced toughness, markedly reduced surface roughness, improved anti-fatigue properties, and a lifespan extension by a factor of two under prolonged cyclic loading with large bending moments [9]. Therefore, SMGT not only eliminates “orange peel” defects but also offers substantial improvements in the strength–ductility trade-off.

Although certain nano-gradient structured materials exhibit superior mechanical properties, the majority of research has predominantly focused on tensile tests. However, these tests may not adequately reflect the behavior of tubes under complex stress conditions during hydroforming. Moreover, the nano-gradient structure is significantly influenced by the process parameters of SMGT, such as rotation speed, feed rate, and cooling rate, which in turn affect the mechanical properties of the tubes [11]. There have been limited studies on the SMGT of aluminum alloy tubes. This study specifically examines 6063-T4 aluminum alloy tubes, commonly utilized as raw materials for forming aluminum alloy subframes in automobiles. SMGT experiments were conducted using three distinct sets of parameters to achieve varying nano-gradient structures along the radial direction of the tube. Hydro-bulging tests were performed to evaluate the mechanical properties of the tubes under biaxial stress states. Microstructure analysis of the nanograins on the outermost surface elucidated the strengthening mechanism of the aluminum alloy tube under different SMGT parameters. The findings from this study provide an experimental reference for the surface nano-crystallization of aluminum alloy tubes and offer valuable insights into enhancing the strength and toughness of integrally formed aluminum alloy tubes.

## 2. Materials and Methods

### 2.1. Surface Mechanical Grinding Treatment for Tubes

**Figure 2:**
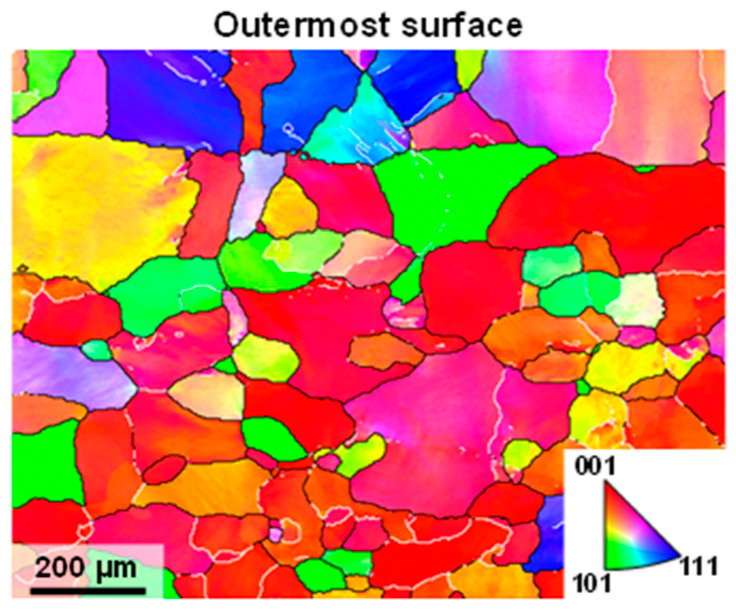
The radial microstructure of the initial tube used for SMGT.

The 6063-T4 aluminum alloy tubes were selected. The chemical composition of the elements contained in the tube is presented in Table 1, and its radial microstructure, characterized by an average grain size of 175 μm, is illustrated in Figure 2.

**Figure 3:**
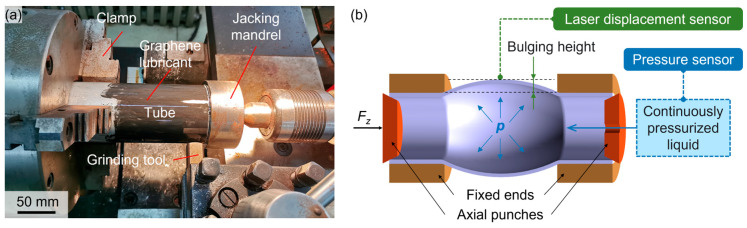
Schematic diagrams: (**a**) illustration of the SMGT device for tubes; (**b**) setup for tube hydro-bulging tests.

The SMGT setup for the tubes using a modified horizontal lathe is illustrated in Figure 3a. One end of each tube was securely clamped, while a jacking mandrel restrained the opposite end axially. A spherical grinding tool, mounted on the tool platform, was employed to grind the outer surface of the tubes. Prior to SMGT, a uniform layer of graphene lubricant was applied to the outer surface of the tubes. The spindle rotation speed was denoted as *ω*. The radial feed rate of the spherical tool was set at 0.5 mm per pass, and the axial feed rate was 5 mm/min. Each tube underwent two passes. Three sets of SMGT experiments were conducted to investigate the effects of process parameters on the mechanical properties of the tubes. In the first set (S1), the spindle rotation speed was 1120 r/min with water cooling; in the second set (S2), it was 710 r/min with water cooling; and in the third set (S3), it was 1120 r/min with air cooling. All process parameters are summarized in Table 2. After SMGT treatment, the wall thickness of the tubes was reduced to approximately 3.8 mm, resulting in an outer diameter of approximately 77.6 mm. The technical drawings of the initial tubes and SMGTed tubes are shown in the Supplementary Materials Figure S1.

### 2.2. Mechanical Property Testing of Tubes

The tube hydro-bulging tests were conducted at the Fluid Pressure Research Institute of Harbin Institute of Technology to evaluate the mechanical properties of the tubes under biaxial stress states using a specialized tube hydro-bulging performance testing device. As illustrated in Figure 3b, the schematic diagram of the tube hydro-bulging setup shows that the two ends of the tube were restrained by fixed ends to prevent axial movement and sealed by two axial rigid punches. The tube was gradually expanded by a pressurized liquid inside. Before the bulging process, it is necessary to measure the initial thickness of the tube, its initial outer diameter, and the length of the bulging zone. During the bulging process, a laser displacement sensor records the displacement of the highest point on the tube, while a pressure sensor monitors the internal liquid pressure. Through the linear model of pole thickness and bulging height, it is possible to derive the equivalent strain, the radius of curvature in both circumferential and axial directions, as well as each stress component [12]. Stress–strain components were calculated to establish the stress–strain relationship of the tube through a linear model for pole thickness change [13]. Yield strengths were defined as the stress value corresponding to a 0.2% offset strain on the engineering stress–strain curve. Three tube specimens for each parameter were utilized in the experiment, and the specimen with the median yield strength was selected as the representative result. Micro-hardness measurements, performed using Vickers hardness testing with an applied force of 100 g and a holding time of 10 s, characterized the hardening behavior of the cross-section. At least 10 points were measured for each specimen.

### 2.3. Microstructure Characterization

The axial direction of the tubes was defined as AD, the radial direction as RD, and the grinding direction as GD. The regions near the top surface of the RD-AD cross-section were polished using an argon ion beam and subsequently characterized by backscattered electron (BSE; Oxford C-nano, Oxford, UK) imaging to examine overall morphologies and quantify the thickness of the gradient layer. Additionally, micro-hardness profiles across the RD-AD sections were measured to delineate the hardness-affected zone. To elucidate the strengthening mechanisms in SMGTed tubes, three foil specimens were prepared from the top surface along the RD-GD sections using focused ion beam (FIB; Tescan Amber, Brno, Czech Republic) milling for detailed analysis of grain morphology and substructure characteristics. Prior to FIB milling, platinum was deposited on the surface to prevent damage. The FIB-prepared specimens were characterized by transmission electron microscopy (TEM; Talos F200X, Thermo Fisher, Waltham, MA, USA) operating at an accelerating voltage of 200 kV. The thickness of the foils was quantitatively estimated using convergent beam electron diffraction techniques. The weak-beam dark-field (WBDF) TEM technique was employed to observe dislocations in each region [14]. The line intersection method was used to determine the dislocation densities of the different specimens. This involved the overlay of a grid of horizontal (length $L_{h}$) and vertical (length $L_{v}$) lines over a selected area (which contained dislocations) and the counting of the intersections ($n_{h}$ and $n_{v}$) of these lines with the dislocations to calculate the dislocation density as [15].

(1) $$\rho =\left(\frac{n_{h}}{L_{h}}+\frac{n_{v}}{L_{v}}\right)/t$$

where t denotes the thickness of the observation area. Furthermore, the fracture morphologies of bulged tubes were examined using a scanning electron microscopy (SEM; Tescan Amber, Brno, Czech Republic).

## 3. Results

### 3.1. Mechanical Properties of Tubes Under Biaxial Stress States

**Figure 4:**
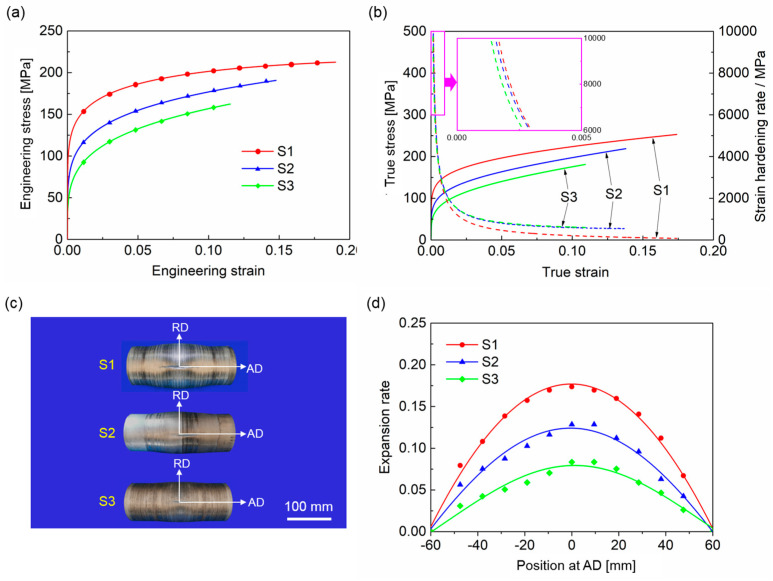
(**a**) Engineering stress–strain curves, (**b**) true stress–strain and strain hardening curves under biaxial stress states of SMGTed tubes; (**c**) hydro-bulged SMGTed tubes and their (**d**) expansion rate distributions.

Among the stress–strain curves of the three groups of specimens, the curve corresponding to the intermediate yield strength of each group was selected as the representative result for inter-group comparison. Figure 4a presents the engineering stress–strain curves of the three groups of specimens under biaxial stress states. The yield strengths of S1, S2, and S3 decrease sequentially to 120 MPa, 91 MPa, and 67 MPa, respectively. The stress exhibits a gradual increase with increasing strain. At engineering strains of 0.189, 0.148, and 0.116 for S1, S2, and S3, respectively, the stress reaches peak values of 208 MPa, 191 MPa, and 163 MPa. The corresponding true stress–strain and strain-hardening curves are illustrated in Figure 4b. In the initial stage of bulging, S1 demonstrates the highest strain-hardening rate, followed by S2. Once the strain surpasses 0.005, the strain-hardening rate of S1 becomes lower than that of the other two specimens. Compared to S2 and S3, S1 shows more sustainable strain hardening. The strain hardening of S2 is marginally more sustained compared to S3. Based on the Considère criterion, Equation (1), the necking instability will occur when the strain hardening rate drops to the value of the flow stress. The ductility of the tube is expressed by the ultimate equivalent strain [16], as follows:

(2) $$\frac{d\sigma_{T}}{d\varepsilon_{T}}=\sigma_{T}$$

where the true strain $\varepsilon_{T}$ represents the ultimate equivalent strain before instability under biaxial stress states. Hence, the ductility of S1, S2, and S3 decrease in that order.

Figure 4c displays the tubes post hydro-bulging, while Figure 4d presents the expansion rates at different axial positions, which indicate an increase in circumference. The expansion rate peaks in the middle of the tube and gradually decreases toward both ends, leading to crack formation near the center. These cracks develop along the axial direction, suggesting excessive circumferential stress that results in fractures. The maximum expansion rate of the tube specimens reflects their formability under biaxial stress conditions and serves as an indicator of their ductility [17,18]. It is noted that among the specimens, S1 exhibits superior formability, followed by S2, with S3 showing the least formability. The corresponding maximum expansion rates and mechanical properties are summarized in Table 3. It is clear that S1 possesses high strength and the best formability, whereas S3 has the lowest strength and worst formability. Among the three specimens, S1 exhibits both the highest strength and the best ductility. In contrast, S3 demonstrates the lowest strength and poorest ductility.

### 3.2. Microhardness Distributions at Cross-Sections

**Figure 5:**
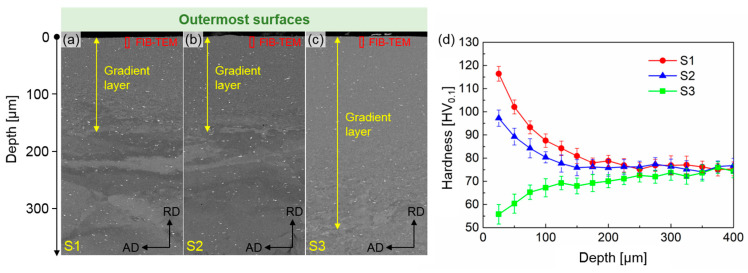
Cross-sectional BSE images of (**a**) S1, (**b**) S2, (**c**) S3, and (**d**) corresponding microhardness distributions.

The BSE images in Figure 5a–c depict the RD-AD cross-sectional views of the SMGTed aluminum alloy tubes. Significant grain refinement is observed outside the tubes, leading to the formation of a gradient layer with a specific thickness [19]. The gradient layer thickness for S1 and S2 is approximately 180 μm, attributed to their faster cooling rates. In contrast, S3 exhibits a thicker gradient layer exceeding 300 μm due to its relatively slower cooling rate. Beyond the gradient layer, the region consists of coarse grains, including both deformed and undeformed coarse grains [20]. Figure 5d illustrates the corresponding mechanical properties, specifically the Vickers hardness distribution along the depth of the cross sections. Clearly, a gradient hardness distribution is evident within the gradient layer of the specimens. For S1 and S2, which were prepared under water cooling, the hardness gradually increases from the interior toward the surface, reaching peak values of 117 HV and 98 HV, respectively. Conversely, S3, fabricated under air cooling, shows a gradual decrease in hardness from the interior to the surface, with a minimum hardness of 56 HV. The hardness in the internal regions of all three specimens converges at 75 HV, as these areas remain unaffected by the mechanical grinding treatment [21]. To further elucidate the influence mechanism of the SMGT process on the mechanical properties of naturally aged aluminum alloy tubes, foil specimens were extracted from the outermost surfaces of the SMGT-treated tubes via FIB for microstructural observation, as indicated by the red boxes as shown in Figure 5a–c.

### 3.3. Microstructures at the Outermost Surface

**Figure 6:**
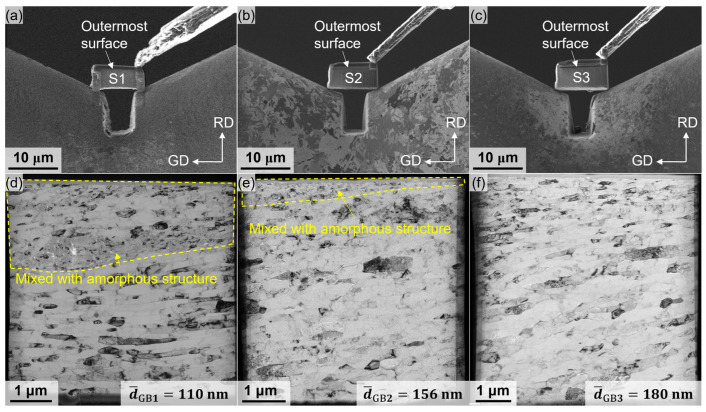
Foil specimens acquired from the outermost surface of SMGTed tubes (**a**) S1, (**b**) S2, (**c**) S3, and the corresponding overall grain morphologies (**d**) S1, (**e**) S2, and (**f**) S3.

Figure 6a–c displays the foil specimens extracted from the outermost surfaces of SMGTed tubes S1, S2, and S3, while Figure 6d–f illustrates their overall morphologies. A region mixed with amorphous structure [22] is observed near the outermost surfaces of both S1 and S2 foil specimens, as indicated by the yellow dotted lines but not in S3. Apart from this amorphous region, the grains in all three specimens are elongated along the GD direction and exhibit nano-lamellar structures. However, the lamellar spacings vary among the specimens. Specifically, S1, prepared under water cooling conditions and a relatively high grinding speed of 1120 r/min, exhibits the smallest lamellar spacing with an average value of 110 nm. S2, also prepared under water cooling conditions but at a lower grinding speed of 720 r/min, shows a larger lamellar spacing. Despite having the same grinding speed as S1 (1120 r/min), S3 prepared under air cooling conditions leads to the largest lamellar spacing of 180 nm.

### 3.4. Fracture Morphologies of Bulged Tubes

**Figure 7:**
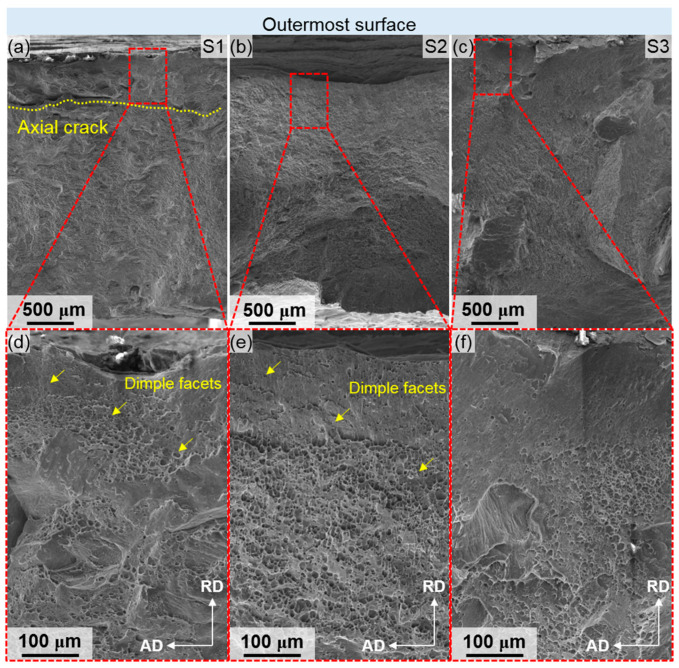
Fracture morphologies of bulged tubes (**a**) S1, (**b**) S2, (**c**) S3, and their enlarged images (**d**) S1, (**e**) S2, and (**f**) S3.

The fracture morphologies of the hydro-bulging tubes are illustrated in Figure 7. In the fracture of S1, a visible axial crack is observed near the gradient layer. Conversely, no axial cracks are evident in the fractures of S2 and S3. The presence of an axial crack in S1 indicates inconsistent deformation between the gradient layer and the coarse-grained region under biaxial tensile stress, leading to mutual resistance. By contrast, the tube specimens of S2 and S3 do not exhibit significant inconsistent deformation upon fracturing, allowing the cracks to propagate rapidly. A closer examination of the enlarged morphology of S1 reveals numerous equiaxed dimple facets (indicated by the yellow arrows) in the region corresponding to the gradient layer, with some intergranular smooth facets gradually forming inward. In the enlarged morphology of S2, fewer equiaxed dimple facets are present on the outermost surface, while more such facets appear inward. Additionally, in the enlarged morphology of S3, a large smooth intergranular facet is observed near the outermost surface, with some dimple facets forming approximately 200 μm inward from the surface.

## 4. Discussion

### 4.1. Microstructure of SMGTed Aluminum Alloy Tubes

**Figure 8:**
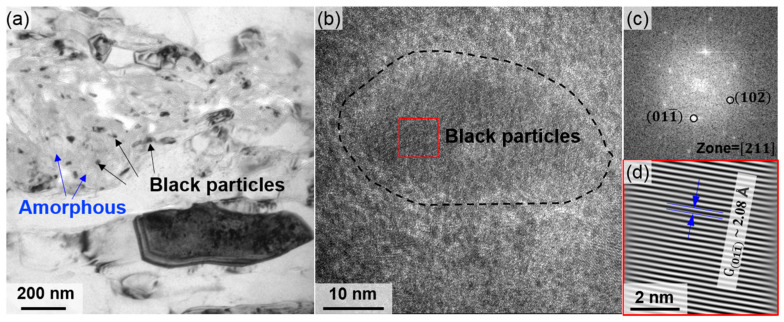
(**a**) Bright-field TEM image showing the area containing amorphous structures and lamellar grains of S1; (**b**) HRTEM image showing the black particles, its (**c**) FFT, and (**d**) inverse FFT patterns.

As an effective method of severe plastic deformation, SMGT induces significant grain refinement on the surface of aluminum alloy tubes [23]. Obvious gradient layers formed near the surface of the tubes. The thicknesses of the gradient layers in S1 and S2 were similar (Figure 4b and Figure 5a), whereas the thickness of the gradient layer in S3 was significantly larger (Figure 5c). The outermost surfaces of S1, S2, and S3 are refined from the original 175 μm to nanoscale lamellar grains, with lamellar spacings of 110 nm, 156 nm, and 180 nm, respectively (Figure 6). Additionally, amorphous regions are observed on the outermost surfaces of S1 and S2, with the area of the amorphous region in S1 being marginally larger than that in S2. In the bright-field TEM images of these amorphous regions, numerous black particles, ranging in size from 10 to 50 nm, are found surrounding the amorphous structures (Figure 8a). The high-resolution transmission electron microscopy (HRTEM) image of the black particles in Figure 8b, along with the corresponding fast Fourier transform (FFT) in Figure 8c and inverse FFT patterns in Figure 8d, confirm an interplanar spacing of 2.08 Å in the $01\bar{1}$ direction, indicating the characteristics of graphene. Graphene is found on the outermost surfaces of S1 and S2 rather than in S3. The presence of graphene facilitates acting as heterogeneous nucleation sites during SMGT, prompting the formation of amorphous structures in the surrounding Al matrix under high strain and strain rates [24]. Therefore, in S1 and S2, moving from the outermost surface to the inner region of the tube, the amorphous region, the nano-gradient layer, and the coarse-grain region are observable sequentially. In S3, only the nano-gradient layer and the coarse-grain region are present in sequence. No amorphous structure was observed in S3 due to its relatively slow cooling rate. The amorphous structure formed by severe plastic deformation quickly transitions into a crystalline state under the influence of frictional heat.

### 4.2. Precipitate Behavior in SMGTed Aluminum Alloy Tubes

**Figure 9:**
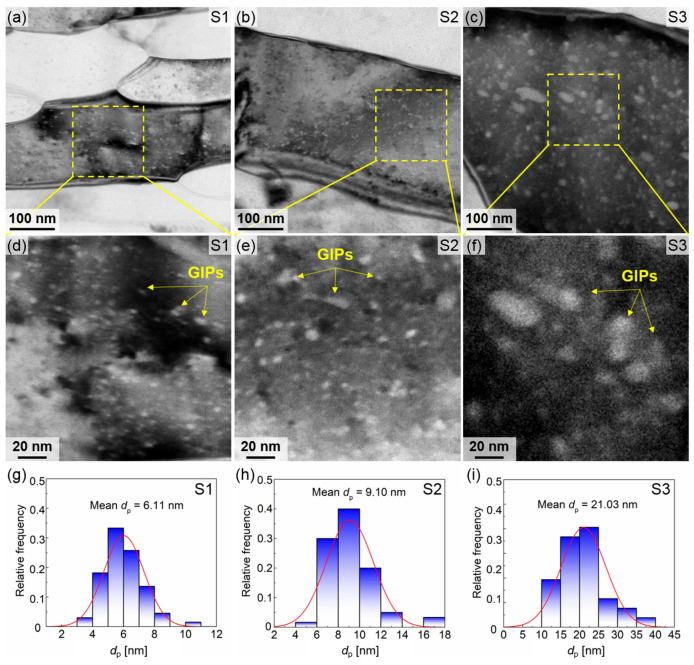
Distributions of GIPs for the three foil specimens: (**a**–**c**) bright field TEM images, (**d**–**f**) locally enlarged images, and (**g**–**i**) their size distributions.

Numerical nanoscale grain internal precipitations (GIPs) are observed in S1, S2, and S3. This indicates that precipitate redistribution occurs on the surface of the tube during the SMGT process [25]. Bright-field TEM images of the GIPs from the three specimens are shown in Figure 9a–c, with enlarged views provided in Figure 9d–f. It is evident that the GIPs are uniformly distributed within the grains and predominantly rod-shaped. The sizes of GIPs in S1 and S2 are similar due to their preparation under water cooling conditions. However, because of the higher strain rate experienced by S1 compared to S2, the GIPs in S1 are smaller than those in S2. S3, prepared at room temperature with a relatively slower cooling rate, exhibits significantly larger GIPs. The equivalent diameter is utilized to quantify the size of GIPs, and the frequency distribution of these diameters for the GIPs in the three specimens is illustrated in Figure 9g–i. The mean sizes of GIPs are 6.11 nm for S1, 9.10 nm for S2, and 21.03 nm for S3. The number densities of GIPs were also determined based on unit volume statistics presented in Figure 9g–i, revealing a highest density of 9.49 × 10^22^ m^−3^ in S1, a moderately higher density of 6.25 × 10^22^ m^−3^ in S2, and the lowest density of 1.74 × 10^22^ m^−3^ in S3.

**Figure 10:**
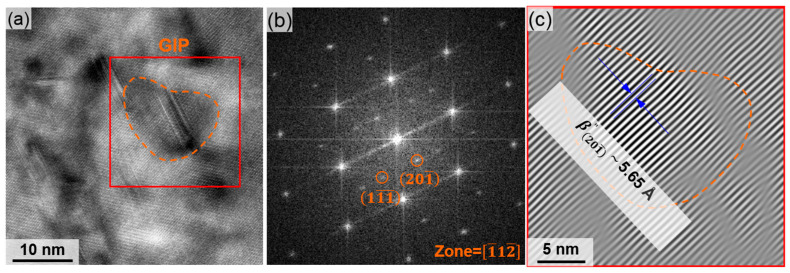
GIP at the outermost surface of S1 and S2: (**a**) HRTEM image, its (**b**) FFT, and (**c**) inverse FFT patterns confirming the existence of $\beta^{”}$.

The GIPs in S1 and S2 are determined to be $\beta^{”}$. The HRTEM image is shown in Figure 10a, with corresponding FFT and inverse FFT patterns presented in Figure 10b,c, revealing an interplanar spacing of 5.65 Å along the [$20\bar{1}$] direction. Diffraction analysis confirmed that most GIPs are $\beta^{”}$(Mg_5_Si_6_) [26], exhibiting a monoclinic structure aligned with the Al matrix through Guinier–Preston zones [27]. The $\beta^{”}$ phase, characterized by its needle-like morphology and monoclinic structure, is specifically oriented relative to the Al matrix. This phase is known to significantly enhance hardness and serves as a critical strengthening component in 6000-series aluminum alloys [28].

.

**Figure 11:**
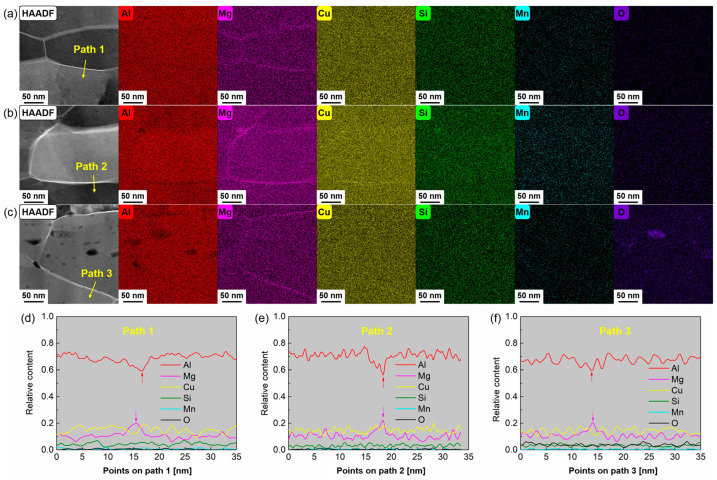
HAADF and EDS maps of the principal elements (i.e., Al, Mg, Cu, Si, Mn, and O) near the grain boundaries for the three foil specimens: (**a**) S1, (**b**) S2, and (**c**) S3; line scans of elemental distributions along paths perpendicular to the grain boundaries: (**d**) S1, (**e**) S2, and (**f**) S3.

However, GIPs in S3 not only contain $\beta^{”}$ phases but also include oxides that are tens of nanometers in size. This observation is supported by the High Angle Annular Dark Field (HAADF) images and corresponding Energy Dispersive Spectrometer (EDS) maps shown in Figure 11a–c. The formation of these oxides can be ascribed to the slow cooling rate during severe plastic deformation. The friction-induced heat generation, which cannot be dissipated promptly, increases atomic activity and promotes their reactivity with oxygen in the ambient environment. This facilitates oxidation and results in the development of an oxide layer tens of nanometers in thickness. Figure 11d–f illustrates the relative elemental content along specific path lines for three specimens, with arrows indicating the positions of the grain boundaries. The data reveal a higher oxygen content in S3 compared to the other two specimens. The presence of these oxides hinders the formation of GIP within the grains, leading to reduced strength and ductility in the S3 tube relative to other types of tubes [29]. Additionally, the precipitation behavior of GIPs on the tube surface is influenced by elemental segregation [30]. Notably, Mg segregation at the grain boundaries of S2 is significantly higher than that of S1. Given that Mg is a primary constituent of $\beta^{”}$, this increased segregation results in Mg depletion within the grains of S2, thereby affecting $\beta^{”}$ formation.

### 4.3. Strengthening Mechanisms at the Outermost Surface of SMGTed Tubes

On the outermost surface of SMGTed tubes, S1 exhibits the highest hardness, followed by S2, while S3 demonstrates the lowest hardness, even lower than that of the original coarse-grained region, as shown in Figure 5d. The variation in hardness can be attributed to the evolution of microstructure. The primary strengthening mechanism is grain boundary strengthening [31]. Specifically, the grains at the outermost surface are significantly refined, transitioning from equiaxed grains with an average size of 175 μm to lamellar grains with spacings of 110 nm, 156 nm, and 180 nm, respectively. The strength increment due to grain refinement can be obtained from the Hall–Petch relationship [32],

(3) $$∆\sigma_{gb}=k∆d_{gb}^{-\frac{1}{2}}$$

where $∆\sigma_{gb}$ represents the strength increment due to grain refinement, $d_{gb}$ is the lamellar spacing, and *k* is the Hall–Petch coefficient, which is 60 MPa μm^1/2^ for several Al-based alloys [33]. The contributions of grain refinement strengthening are approximately 176, 147, and 136 MPa, based on the dimensional change for the outermost surface in S1, S2, and S3, respectively.

Secondly, amorphous regions are observed on the outermost surfaces of both S1 and S2. Although these regions cover relatively small areas (approximately 2 μm in width for S1 and approximately 500 nm for S2, see Figure 6d,e), amorphous structures are typically considered to exhibit high strength and low ductility [22], which can contribute to enhancing the strength of tubes. However, during the tube bulging process, plastic deformation occurs on the inner surface earlier than on the outer surface. Consequently, the presence of a small amorphous region on the outer surface has minimal impact on the overall ductility.

Thirdly, precipitation strengthening constitutes a critical mechanism for enhancing the outermost surface of S1 and S2. Notably, a substantial presence of the $\beta^{”}$ phase is observed in both S1 and S2, which is a well-known strengthening phase in 6000 series aluminum alloys. The Orowan equation can be used to estimate the contribution of precipitation strengthening [33], as follows:

(4) $$∆\sigma_{p}=M\frac{0.4Gb}{\pi \sqrt{1-\upsilon }}\frac{ln\left(\bar{d}/b\right)}{\lambda_{p}}$$

where M is the Taylor factor 2.94, b is the Burgers vector 0.286 nm, υ is the Poisson’s ratio which is 0.345 for Al [34], G is the shear modulus, which is 26.38 GPa for the AlMgSi alloy [35], $\bar{d}$ is the average diameter of the nano-precipitations, and $\lambda_{p}$ is the average distance between neighboring the nano-precipitations. Therefore, the contribution of precipitation strengthening to the outermost surface of S1 is marginally greater than that of S2. However, the presence of oxides on the outermost surface of S3 significantly diminishes its strength while increasing its brittleness.

**Figure 12:**
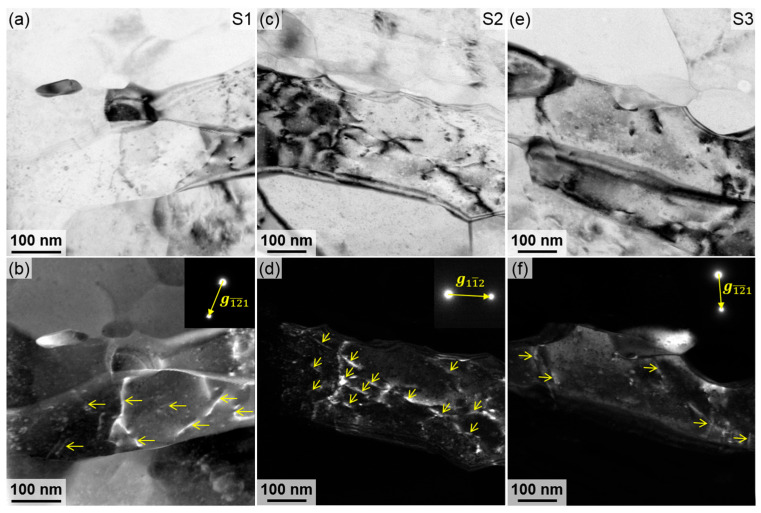
Bright-field and WBDF images showing the intragranular structural features related to the dislocations at the outermost surface of the three foil specimens (**a**,**b**) S1; (**c**,**d**) S2; and (**e**,**f**) S3. (The method of WBDF is based on [14]).

Finally, dislocation hardening constitutes a critical strengthening mechanism for the outermost surface of tubes. Figure 12 illustrates the intragranular structural features associated with dislocations at the outermost surface, depicted as yellow lines under the <112> *g*-vectors. According to Equation (1), the dislocation densities in S1, S2, and S3 are calculated to be 3.49 × 10^14^ m^−2^, 6.04 × 10^14^ m^−2^, and 2.38 × 10^14^ m^−2^, respectively, based on measurements from foils with a thickness of approximately 65 nm. Dislocation hardening can be calculated by the Taylor equation [36], as follows:

(5) $${∆\sigma }_{dis}=M\alpha bG\sqrt{\rho }$$

where α is a constant (0.24) and ρ is the total dislocation density. It is evident that dislocation hardening has the most pronounced effect on S2, followed by S1, and the least on S3. This phenomenon can be attributed to the slower cooling rate of the S3, which facilitates the movement and annihilation of dislocations within the grains [10]. Although S1 and S2 experience the same cooling rate, S1 rotates faster and generates more heat compared to S2. This increased heat promotes the migration of some dislocation lines to the grain boundaries and enhances dynamic recovery [37], resulting in fewer dislocation lines accumulating within the grains.

### 4.4. Deformation Mechanism of SMGTed Tubes During Hydro-Bulging

Based on the tube hydro-bulging tests, S1 demonstrates the highest strength and best ductility, followed by S2, while S3 exhibits the poorest performance. The primary microstructural difference among the three specimens is the gradient layer on the outer surface. Specifically, the outermost surface of S1 features an approximately 2 μm-thick amorphous region and lamellar grains with a spacing of 110 nm, which significantly enhances its strength. These nano-lamellar grains contain a high density of GIP ($\beta^{”}$), not only boosting the strength but also facilitating dislocation accumulation during deformation, thereby improving ductility [5]. As a result, S1 exhibits a high strain hardening rate at the initial bulging stage. However, due to the limited size of the nanograins and their restricted ability to accumulate dislocations [38], the strain hardening rate decreases rapidly after the strain exceeds 0.02. Despite this, S1 maintains the most sustained strain hardening capability throughout the entire process, leading to the highest ultimate strain and maximum expansion rate, thus exhibiting superior ductility. Consequently, the outermost surface of the tube shows significant resistance during the fracture process (Figure 7).

Compared with S1, the strength and ductility of S2 are both inferior due to a smaller amorphous region, larger lamellar spacing, and a reduced amount of GIP ($\beta^{”}$). The lower density of GIP ($\beta^{”}$) diminishes its contribution to precipitation strengthening, limiting dislocation accumulation and resulting in a less effective strengthening effect and poorer ductility. Among the three specimens, S3 exhibits the worst strength and ductility, primarily due to the presence of numerous oxides on the outer surface. These oxides, which are typically detrimental phases in the aluminum alloy matrix [39], possess lower strength and higher brittleness compared to the matrix, thereby significantly reducing the overall mechanical properties.

## 5. Conclusions

The 6063-T4 aluminum alloy tubes underwent surface mechanical grinding using various parameters, after which their mechanical properties under biaxial stress conditions were evaluated via tube hydro-bulging tests. Additionally, the cross-sectional hardness distributions were analyzed, and the microstructures of the outermost surfaces were examined. The key findings are summarized as follows.

1. Under graphene lubrication, surface mechanical grinding treatment (SMGT) was applied to the tubes. With water cooling, a gradient layer approximately 180 μm thick was formed, featuring an amorphous outer surface and nano-lamellar grains, along with internal $\beta^{”}$ (Mg_5_Si_6_) precipitates. The density of nano-precipitates increases with higher rotational speeds. In contrast, under air cooling conditions, the gradient layer exceeds 300 μm in thickness, displaying lamellar grains on the outer surface and containing both $\beta^{”}$ precipitates and oxides within its interior;
2. The tube prepared under the condition of 1120 r/min with water cooling exhibits significantly enhanced strength during tube bulging, which can be attributed to grain boundary strengthening, precipitation strengthening, and dislocation hardening. Specifically, the yield strength reaches up to 120 MPa. In contrast, when the rotational speed is reduced to 710 r/min, the lamellar spacing decreases, the size of precipitates increases, and their density decreases. Despite this, the dislocation density increases, ultimately leading to a reduction in overall strength. Moreover, when the tube is fabricated at 1120 r/min with air cooling, oxides form within the lamellar grains, thereby compromising the mechanical strength;
3. The tube, operating at a rotational speed of 1120 r/min under water-cooling conditions, demonstrates a high hardening rate during the initial deformation stage. Although this hardening rate decreases rapidly in subsequent stages, the tube retains a sustained strain hardening capability throughout the entire process. Fracture morphology analysis reveals that the gradient layer plays a critical role in enhancing the tube’s resistance to fracture across its entirety. In contrast, when the rotational speed is reduced to 710 r/min or air cooling is used instead of water cooling, both the sustained strain hardening capacity and ductility of the tubes decline significantly;
4. Surface mechanical grinding treatment can effectively enhance the strength and toughness of aluminum alloy tubes at room temperature. The process parameters of this treatment are directly correlated with the gradient of microstructure and the resulting mechanical properties of the tube. Future research could leverage artificial intelligence to optimize these parameters, thereby fully maximizing the advantages of gradient-structured aluminum alloy tubes.

## Figures and Tables

**Table 1:** Chemical composition of the 6063-T4 aluminum alloy tube [wt.%].

Mg	Cu	Si	Mn	Al
0.60	0.10	0.40	0.10	balanced

**Table 2:** The SMGT parameters for aluminum alloy tubes in the current study.

Specimen No.	Rotation Speed *ω* [r/min]	Lubrication	Cooling	Radial Feed	Grinding Tool Speed
S1	1120	Grease/Graphene	Water-cooling	0.5 mm	5 mm/min
S2	710		Water-cooling		
S3	1120		Air-cooling		

**Table 3:** Mechanical properties of SMGTed tubes under biaxial stress states based on hydro-bulging tests.

Specimen No.	Yield Stress [MPa]	Ultimate Stress [MPa]	Ultimate Strain	Maximum Expansion Rate
S1	120	208	0.189	0.164
S2	91	191	0.148	0.128
S3	67	163	0.116	0.083

## Data Availability

The data presented in this study are available on request from the corresponding author, as the data also form part of an ongoing study.

## Supplementary Materials

The following supporting information can be downloaded at: https://www.mdpi.com/article/10.3390/ma18092038/s1, Figure S1. Technical drawings of the initial tubes and surface mechanical grinding treated (SMGTed) tubes.

## Abbreviations

The following abbreviations are used in this manuscript:

SMGT	Surface Mechanical Grinding Treatment
AD	Axial Direction
RD	Radial Direction
GD	Grinding Direction
BSE	Backscattered Electron
FIB	Focused Ion Beam
TEM	Transmission Electron Microscopy
WBDF	Weak-Beam Dark-Field
SEM	Scanning Electron Microscopy
S1	The First Set
S2	The Second Set
S3	The Third Set
HRTEM	High-Resolution Transmission Electron Microscopy
FFT	fast Fourier transform
GIP	Grain Internal Precipitation
HAADF	High Angle Annular Dark Field
EDS	Energy Dispersive Spectrometer
*ω*	Rotation speed
$L_{h}$	Horizontal length
$L_{v}$	Vertical length
$n_{h}$	Intersections of horizontal lines
$n_{v}$	Intersections of vertical lines
t	Thickness
ρ	Dislocation density
$\varepsilon_{T}$	True strain
$\sigma_{T}$	True stress
*k*	Hall–Petch coefficient
$∆\sigma_{gb}$	Strength increment due to grain refinement
$∆\sigma_{p}$	Strength increment due to precipitation
${∆\sigma }_{dis}$	Strength increment due to dislocation hardening
$d_{gb}$	Lamellar spacing
M	Taylor factor
b	Burgers vector
υ	Poisson’s ratio
G	Shear modulus
$\bar{d}$	Average diameter of the nano-precipitations
$\lambda_{p}$	Average distance between neighboring the nano-precipitations

## References

[1] Yuan S., Fan X. (2019). Developments and perspectives on the precision forming processes for ultra-large size integrated components. Int. J. Extreme Manuf..

[2] Cai Y., Wang X.S., Yuan S.J. (2016). Pre-form design for hydro-forming of aluminum alloy automotive cross members. Int. J. Adv. Manuf. Technol..

[3] Cai Y., Wang X., Yuan S. (2016). Analysis of surface roughening behavior of 6063 aluminum alloy by tensile testing of a trapezoidal uniaxial specimen. Mater. Sci. Eng. A.

[4] Liu X.C., Zhang H.W., Lu K. (2015). Formation of nano-laminated structure in nickel by means of surface mechanical grinding treatment. Acta Mater..

[5] Li X., Lu L., Li J., Zhang X., Gao H. (2020). Mechanical properties and deformation mechanisms of gradient nanostructured metals and alloys. Nat. Rev. Mater..

[6] Nutor R.K., Cao Q., Wei R., Su Q.M., Du G.H., Wang X.D., Li F.S., Zhang D.X., Jiang J.Z. (2021). A dual-phase alloy with ultrahigh strength-ductility synergy over a wide temperature range. Sci. Adv..

[7] Fang T.H., Li W.L., Tao N.R., Lu K. (2011). Revealing Extraordinary Intrinsic Tensile Plasticity in Gradient Nano-Grained Copper. Science.

[8] Wang J.J., Tao N.R., Lu K. (2019). Revealing the deformation mechanisms of nanograins in gradient nanostructured Cu and CuAl alloys under tension. Acta Mater..

[9] Lu K. (2015). Gradient Nanostructured Materials. Acta Metall. Sin..

[10] Ji W., Zhou R., Vivegananthan P., Wu M.S., Gao H., Zhou K. (2023). Recent progress in gradient-structured metals and alloys. Prog. Mater. Sci..

[11] Xu P., Luo H., Han Z., Zou J. (2015). Tailoring a gradient nanostructured age-hardened aluminum alloy using high-gradient strain and strain rate. Mater. Des..

[12] He Z., Yuan S., Lin Y., Wang X.X., Hu W.L. (2014). Analytical model for tube hydro-bulging tests, part II: Linear model for pole thickness and its application. Int. J. Mech. Sci..

[13] He Z., Yuan S., Lin Y., Wang X.X., Hu W.L. (2014). Analytical model for tube hydro-bulging test, part I: Models for stress components and bulging zone profile. Int. J. Mech. Sci..

[14] Zhao D., Xie K. (2021). Dislocation imaging via the virtual dark-field technique using the precession electron diffraction data. Microsc. Microanal..

[15] Wang S., Hashimoto N., Wang Y., Ohnuki S. (2013). Activation volume and density of mobile dislocations in hydrogen-charged iron. Acta Mater..

[16] Zhu Y., Wu X. (2023). Heterostructured materials. Prog. Mater. Sci..

[17] Wang X., Fan X., Chen X., Yuan S. (2022). Cryogenic deformation behavior of 6061 aluminum alloy tube under biaxial tension condition. J. Mater. Process. Tech..

[18] He Z., Zhang K., Zhu H., Lin Y.L., Fu M., Yuan S. (2022). An anisotropic constitutive model for forming of aluminum tubes under both biaxial tension and pure shear stress states. Int. J. Plast..

[19] Oh J., Park H.D., Gwak M., Lee J., Son S., Amanov A., Kim H., Seol J., Sunh H., Kim J. (2021). Mechanical property enhancement in gradient structured aluminum alloy by ultrasonic nanocrystalline surface modification. Mater. Sci. Eng. A.

[20] Hou H., Dong R., Tan Y., Li C., Zhang X., Wu L., Zhu B., Zhao Y. (2023). Microstructural characteristics and enhanced mechanical properties of 2024 aluminum alloy resulting from shot-peening treatment. Mater. Charact..

[21] Meng X., Liu B., Luo L., Ding Y., Rao X., Hu B., Liu Y., Lu J. (2018). The Portevin-Le Châtelier effect of gradient nanostructured 5182 aluminum alloy by surface mechanical attrition treatment. J. Mater. Sci. Technol..

[22] Sahu A., Maurya R.S., Laha T. (2024). Advances in Synthesis and Characterization of Aluminum-Based Amorphous Alloys: A Review. Adv. Eng. Mater..

[23] Li Y., Hou P., Wu Z., Feng Z., Ren Y., Choo H. (2021). Dynamic recrystallization of a wrought magnesium alloy: Grain size and texture maps and their application for mechanical behavior predictions. Mater. Des..

[24] Deng S.Q., Godfrey A., Liu W., Hansen N. (2016). A gradient nanostructure generated in pure copper by platen friction sliding deformation. Scr. Mater..

[25] Du Y., Huo W., Xu J., Zhang W. (2020). Mechanical Behavior and Strengthening Mechanisms in Precipitation-Strengthened Aluminum Alloy with Gradient Structure Induced by Sliding Friction Treatment. Metall. Mater. Trans. A.

[26] Chrominski W., Lewandowska M. (2016). Precipitation phenomena in ultrafine grained Al–Mg–Si alloy with heterogeneous microstructure. Acta Mater..

[27] Kahlenberg R., Wojcik T., Falkinger G., Krejci A., Milkereit B., Kozeschnik E. (2023). On the precipitation mechanisms of β-Mg2Si during continuous heating of AA6061. Acta Mater..

[28] Trink B., Weißensteiner I., Uggowitzer P.J., Strobel K., Hofer-Roblyek A., Pogatscher S. (2023). Processing and microstructure–property relations of Al-Mg-Si-Fe crossover alloys. Acta Mater..

[29] Liao Z., Zhang L., Hou X., Huang X. (2025). Grain boundary segregation of solutes and associated plastic deformation mechanisms in nanocrystalline Al–Cu and Al–Mg alloys: A molecular dynamics study. J. Mater. Res. Technol..

[30] Ma S., Liu W., Li Q., Zhang J., Huang S., Xiong Y., Xu B., Yang T., Zhao S. (2024). Mechanism of elemental segregation around extended defects in high-entropy alloys and its effect on mechanical properties. Acta Mater..

[31] Maleki M., Berndorf S., Mohammadzehi S., Mirzadeh H., Emamy M., Ullmann M., Prahl U. (2023). Grain refinement and improved mechanical properties of Mg-4Zn-0.5Ca-0.5RE magnesium alloy by thermomechanical processing. J. Alloys Compd..

[32] Gu Y., Stiles C.D., El-Awady J.A. (2024). A statistical perspective for predicting the strength of metals: Revisiting the Hall–Petch relationship using machine learning. Acta Mater..

[33] Ma K., Hu T., Yang H., Topping T., Yousefiani A., Lavernia E., Schoenung J. (2016). Coupling of dislocations and precipitates: Impact on the mechanical behavior of ultrafine grained Al–Zn–Mg alloys. Acta Mater..

[34] Mohammadi A., Enikeev N.A., Murashkin M.Y., Arita M., Edalati K. (2021). Examination of inverse Hall-Petch relation in nanostructured aluminum alloys by ultra-severe plastic deformation. J. Mater. Sci. Technol..

[35] Zhang P., Shi K., Bian J., Zhang J., Peng Y., Liu G., Deschamps A., Sun J. (2021). Solute cluster evolution during deformation and high strain hardening capability in naturally aged Al–Zn–Mg alloy. Acta Mater..

[36] Hasan M., Liu Y., An X., Gu J., Song M., Cao Y., Li Y., Zhu Y., Liao X. (2019). Simultaneously enhancing strength and ductility of a high-entropy alloy via gradient hierarchical microstructures. Int. J. Plast..

[37] Li G., Liu C., Ma P., Yang J., Feng Z. (2021). Improving formability and retaining dislocation hardening of heavily cold-worked Al alloy by fast heating and fast deformation. Mater. Sci. Eng. A.

[38] Wu X., Zhu Y., Lu K. (2020). Ductility and strain hardening in gradient and lamellar structured materials. Scr. Mater..

[39] Zhang J., Zhang X., Wei S., Chen X., Pan S., Yang C., Pan H., Zhou D., Zhang D., Qin G. (2025). Deformation faulting in ultrafine-grained aluminum alloys: Nucleation mechanisms and critical assessment of strengthening-ductilization contributions. Acta Mater..

