# Supplementary material for: Mechanical Properties of Aluminum Alloy Tubes Fabricated Through Surface Mechanical Grinding Treatment and Graphene Lubrication Under Biaxial Stress States
Source: Materials (Basel). 2025 Apr 29;18(9):2038. doi: 10.3390/ma18092038 (PMC12072185; doi:10.3390/ma18092038)
Supplement: Supplementary file 1 [file materials-18-02038-s001.zip › materials-3545745-supplementary.pdf]

Supplementary Materials for

Mechanical Properties of Aluminum Alloy Tubes Fabricated Through Surface Mechanical Grinding Treatment and Graphene Lubrication under Biaxial Stress States

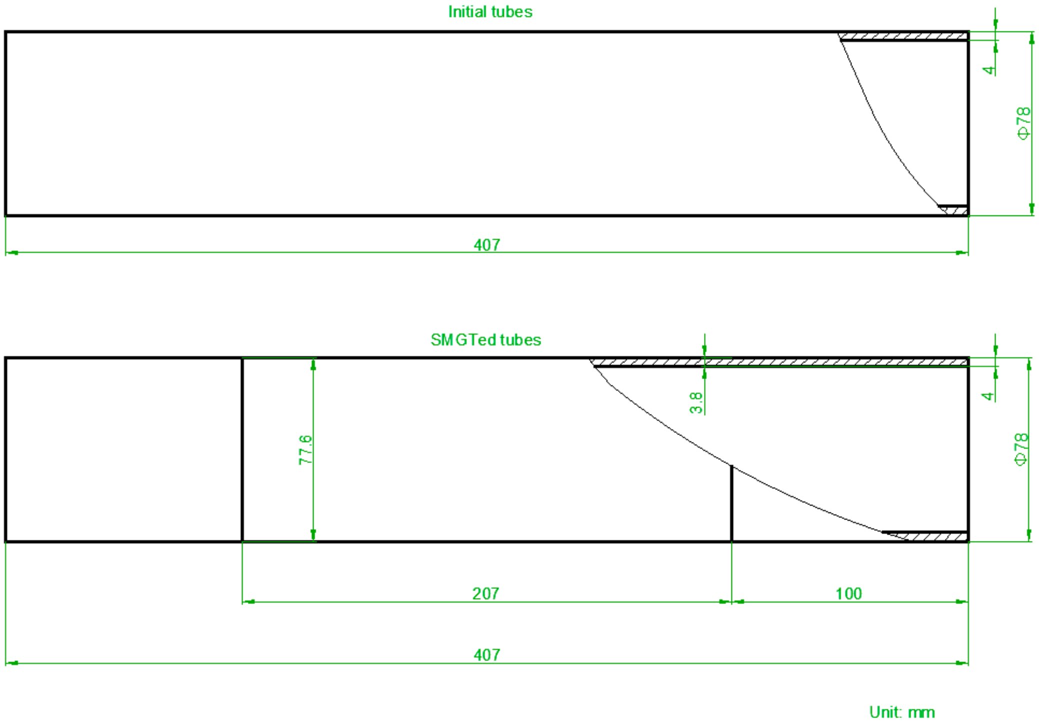

Figure S1. Technical drawings of the initial tubes and surface mechanical grinding treated (SMGTed) tubes.
